# Fecal Eosinophil Cationic Protein Is a Diagnostic and Predictive Biomarker in Young Adults with Inflammatory Bowel Disease

**DOI:** 10.3390/jcm8122025

**Published:** 2019-11-20

**Authors:** Nada Abedin, Teresa Seemann, Sandra Kleinfeld, Jessica Ruehrup, Stefani Röseler, Christian Trautwein, Konrad Streetz, Gernot Sellge

**Affiliations:** 1 Institute for Internal Medicine III, Clinic for Gastroenterology and Hepatology, University Hospital RWTH Aachen, 52074 Aachen, Germany; 2 Department for Internal Medicine I, Gastroenterology, University Hospital Goethe University Frankfurt, 60590 Frankfurt am Main, Germany; 3 Department for Anesthesiology, University Hospital RWTH Aachen 52074 Aachen, Germany; 4 Medical Clinic I, Leverkusen Hospital, 51375 Leverkusen, Germany; 5 Department for Urology, University Hospital Goethe University Frankfurt, 60590 Frankfurt am Main, Germany; 6 Clinic for Dermatology and Allergology, University Hospital RWTH Aachen, 52074 Aachen, Germany; 7 Department for Gastroenterology, Pulmonology and Internal Medicine, Evangelical Hospital Köln-Kalk, 51103 Cologne, Germany; 8 Department for Internal Medicine II, Klinikum Bremen Mitte, St.-Jürgen Str. 1, 28205 Bremen, Germany

**Keywords:** inflammatory bowel disease, biomarker, calprotectin, eosinophils, eosinophil cationic protein

## Abstract

Background and Aims: Fecal biomarkers are important non-invasive markers monitoring disease activity in inflammatory bowel disease (IBD). We compared the significance of fecal eosinophil cationic protein (fECP) and fecal calprotectin (fCal). Methods: fECP and fCal were measured in patients with Crohn’s disease (CD, *n* = 97), ulcerative colitis (UC, *n* = 53), *Clostridioides difficile* infection (CDI, *n* = 9), primary food allergy (PFA, *n* = 11), pollen-associated food allergy (*n* = 25) and non-inflammatory controls (*n* = 78). Results were correlated with clinical and endoscopic IBD activity scores. Results: fECP was significantly elevated in CD, UC, CDI and PFA compared to controls. fCal was significantly increased in CD, UC and CDI. fECP had lower diagnostic accuracy than fCal (area under the curve (AUC) = 0.88) in differentiating between endoscopically active and inactive patients with IBD (AUC = 0.77, ROC analysis). In contrast to fCal, fECP correlated negatively with age and levels were also elevated in clinically and endoscopically inactive patients with IBD <45 years (endoscopically inactive IBD vs controls; AUC for fECP = 0.86; AUC for fCal = 0.62). However, in those patients with low inflammatory activity (fCal <250 mg/kg), high fECP indicated the need for treatment modification or surgery (fECP <200 µg/kg = 22%; 200–600 µg/kg = 44%; >600 µg/kg = 82%) at month 48 of follow-up. Conclusions: fECP is a diagnostic and prognostic marker in young patients with IBD in remission.

## 1. Introduction

Endoscopy is the gold standard for the diagnosis and determination of disease activity in inflammatory bowel disease (IBD). In addition to this invasive procedure, biomarkers have been introduced to monitor disease activity in IBD [1].

Inflammatory markers in the blood, such as C-reactive protein (CRP), indicate systemic inflammation in severely active IBD and disease complications such as abscess formation and bowel perforation [2,3]. Their limitation is both low sensitivity in mildly and moderately active IBD [4] and low specificity, since they cannot differentiate between IBD activity and other forms of inflammation.

Several neutrophil-derived proteins such as calprotectin, lactoferrin, myeloperoxidase and neutrophil elastase have been studied as fecal biomarkers in IBD [5,6]. The most studied fecal biomarker calprotectin (fCal) has high sensitivity and specificity, differentiating active IBD from non-inflammatory intestinal diseases such as irritable bowel syndrome (IBS) [7,8]. Furthermore, fCal is a useful marker for monitoring disease activity [9,10], response to therapy [11] and post-operative recurrence [12,13] as well as for predicting disease relapse in patients in clinical remission [14,15,16,17,18,19]. Nonetheless, fCal is not specific for IBD and is also elevated in other inflammatory diseases of the gut including microscopic colitis [20], gastrointestinal infections [21], diverticulitis [22,23], or colon cancer [23]. fCal is not elevated in the majority of patients with IBD in remission [10,18]. Therefore, fCal cannot be used as a diagnostic marker during remission.

Serum antibodies directed against autoantigens such as perinuclear anti-neutrophil cytoplasmic antibodies (pANCA) or directed against bacterial antigens such as anti-*Saccharomyces cervisiae* antibodies (ASCA) have been studied for diagnosis and disease stratification in patients with IBD. The combination of pANCA and ASCA has a moderate sensitivity and specificity in differentiating patients with Crohn’s disease (CD), ulcerative colitis (UC) and individuals without IBD [24]. Furthermore, the presence of ASCA in patients with CD is associated with a higher risk of developing strictures or penetrating disease phenotypes and of requiring surgery [25,26]. pANCA and ASCA are independent of current disease activity, which is an advantage for their use in inactive patients with IBD. However, their general utility is hampered by the relatively low diagnostic accuracy [27]. 

Activated eosinophilic granulocytes are frequently found in patients with IBD [28]—eosinophil-associated genes are correlated with IBD [29], suggesting that eosinophils contribute to chronic intestinal inflammation in IBD. Eosinophils are present in physiological conditions throughout the gastrointestinal tract distal to the squamous oesophagus [30], whereas they are indicators of pathological conditions at most other tissue sites. Increased numbers of eosinophils in the gastrointestinal tract are found in primary eosinophilic diseases such as eosinophilic esophagitis, gastroenteritis and colitis, as well as secondary conditions such as allergies, infectious diseases, celiac disease and IBD [28,30,31].

Tissue-resident eosinophils are activated in inflammatory diseases of the gastrointestinal tract such as IBD [28] and others such as microscopic colitis [32]. After activation, eosinophils release preformed granular proteins and de novo produced lipid mediators and cytokines [33]. Eosinophil granular proteins such as eosinophil cationic protein (ECP), eosinophil protein X (EPX) or eosinophil-derived neurotoxin are also released in the intestinal lumen and can be detected in feces. ECP, which was studied here, belongs to the ribonuclease family. ECP plays a role in RNA metabolism, possesses bactericidal and helmintho-toxic activity and induces host cell apoptosis and necrosis [33,34].

Elevated fecal ECP (fECP) and fEPX levels in patients with IBD were found in a few studies with small cohorts [35,36,37,38,39,40,41]. They are stable at room temperature for several days, thus fulfilling the requirements of convenient biomarkers [35,38]. However, a careful correlation of fecal eosinophil-derived markers combined with endoscopic activity scores and well-established biomarkers, such as fCal, has not yet been performed.

Both neutrophil- and eosinophil-derived proteins are significantly elevated in the feces of patients with IBD. This led to the hypothesis that the combination of the two markers could better predict the state of inflammation and disease progression in those patients.

Given that eosinophil-derived proteins are frequently elevated in diseases in young patients, especially children, our second hypothesis was that fECP could potentially be a differentiating marker in younger patients with IBD.

Therefore, we analyzed fECP and fCal in 212 samples from 150 patients with IBD as well as in additional healthy controls and patients with IBS, food allergies and *Clostridioides difficile* infection (CDI). fECP and fCal levels were compared among different patient cohorts and were correlated with disease activity markers including endoscopy, disease phenotype, demographic data and disease progression in patients with IBD.

## 2. Materials and Methods 

### 2.1. Patient Selection

Patients were recruited between 2013 and 2015 from the Department of Gastroenterology and Department of Dermatology and Allergology at University Hospital RWTH Aachen during regular outpatient visits or inpatient treatment. Stool and blood samples, as well as clinical data, were collected from previously diagnosed patients with CD, UC, IBS, CDI, primary food allergy (PFA), secondary pollen-associated food allergy (SFA) as well as control patients without intestinal disease (disease control (DC)) and healthy controls (HC). Detailed patient characteristics can be found in Table 1 and Table 2. All patients were above the age of 18 years and signed a written informed consent. Exclusion criteria were current infections, presence of a stoma or ileoanal pouch or pregnancy. 

Irritable bowel syndrome was diagnosed according to the guidelines of the German association of digestive and metabolic disease (DGVS) [42]. 

Food allergy was diagnosed in patients with typical clinical symptoms including local reactions such as oral allergy syndrome, nausea, vomiting, abdominal pain, diarrhea and/or acute systemic reactions with symptoms in various organ systems (skin and mucosa, respiratory tract, cardiovascular system, gastrointestinal tract) occurring shortly after ingestion of a culprit food and additionally confirmed with a positive skin prick test and/or serum sIgE test for at least one symptom-causing food item. Isolated abdominal symptoms such as bloating, flatulence and constipation were not considered to be specific for food allergies. In 30 out of 36 food-allergic patients, an extensive specific IgE serum screening by the ISAC array (Thermo Fisher Scientific GmbH, Schwerte, Germany) was performed. The ISAC array measures 112 recombinant or purified components of 51 allergen sources. Patients were classified as primary food allergy (PFA) if they were sensitized to at least one allergen with a suggested intestinal sensitization route (wheat *n* = 1; seafood *n* = 3; walnut *n* = 2; milk *n* = 1; kiwi/act d2 *n* = 1, lipid transfer proteins of food origin *n* = 5). Secondary food allergy was diagnosed in patients who were only sensitized to pollen cross-reactive food allergens. 

### 2.2. Fecal Sample Preparation and fECP and fCal Measurement

Stool samples were collected from the patients on the day of the visit or one day before and stored immediately at −80 °C until the day of the analysis. fCal was measured using the ELISA kit RIDASCREEN calprotectin by r-biopharm according to the manufacturer’s instructions. Samples were prepared with the buffer provided in the kit.

fECP was measured in supernatants using the human ECP ELISA Kit according to the manufacturer’s instructions (Aviscera Bioscence, Santa Clara, CA, USA). Fecal extraction was performed on ice, using an eluate buffer containing 1 mg of trypsin inhibitor, 200 µL of Roche Complete Protease Inhibitor EDTA-free stock solution and 10 mL of 50 mM phosphate buffer. The eluate buffer was added to the feces in Eppendorf tubes in a 1:5 ratio followed by extensive vortex mixing and centrifugation (10,000× *g* for 10 min). All samples were measured at a dilution of 1:250. Measurement results above the applied standard curve were repeated using a new sample aliquot with higher dilutions up to 1:2500. 

It has been shown that fECP is stable for several days in fecal samples at room temperature, just as fCal [35,38]. We confirmed this result by analyzing 5 fecal samples—of which, one part was frozen on the day of sampling and one part one day later. The mean fECP levels of the day 1 samples were 101% of the levels in samples that were immediately frozen. 

According to the manufacturer, the intra-assay variation is 4%–8% coefficient of variation (CV) and the inter-assay variation is 8%–12% CV for serum and plasma samples. In our study, the intra-assay variation for fECP was 11.4 ± 9.4% CV (mean ± SD; *n* = 16 samples measured in duplicates) and 8.8% (median). Further analyses were performed in single measurements.

### 2.3. Clinical Data and Follow up

Clinical questionnaires were completed by all patients and treating physicians to assess clinical activity scores, the Harvey–Bradshaw Index (HBI) for CD and the Simple Clinical Colitis Activity Index (SCCAI) for UC [1]. Montreal classification and disease behavior [1] were defined for all patients with IBD using previously performed imaging, as well as endoscopic results. 

Endoscopic evaluation was performed by experienced gastroenterologists during routine checkups for patients with IBD. Endoscopy was performed in 83 patients with IBD (CD *n* = 42; UC *n* = 41). In patients with CD, a total colonoscopy was performed, and in patients with UC, a total or a partial (at least till descending colon) colonoscopy was performed.

Laboratory results were masked from all participating physicians. Additionally, endoscopic scores were reviewed retrospectively by a second experienced gastroenterologist, who was also blinded from laboratory findings. Scores that were used for the analysis were the Mayo endoscopic subscore for UC and the Simple Endoscopic Score for CD (SES-CD) [1].

Follow-up data were collected from the medical reports. As a marker for disease progression, we analyzed the time to first treatment modification (start or change of immunosuppressants or biologics) or surgery. Furthermore, in patients with a baseline fCal of <250 mg/kg the time to the first fCal measurement ≥250 mg/kg was documented. fCal measurements were performed as clinically required (for further details see results section).

### 2.4. Statistical Analysis 

Statistical analysis was performed using SPSS 25.2., Microsoft Excel and Graphpad Prism 7. Descriptive statistics and appropriate statistical tests were used as described in the figure legends. 

### 2.5. Ethical Statement 

All recruited patients were informed about the aims of the study and signed an informed consent for their participation. Stool samples were stored in the RWTH centralized Biomaterial Bank Aachen (RWTH cBMB, Aachen, Germany) and provided at time of measurements. They were used in accordance with the regulations of the Biomaterial Bank and the approval of the ethics committee of the medical faculty at RWTH Aachen University project approval EK 049/12. 

## 3. Results 

### 3.1. Study Cohort

In total, 69 fecal samples from 53 patients with ulcerative colitis (UC) and 143 fecal samples from 97 patients with Crohn’s disease (CD) were analyzed. Because of the retrospective design of the study with random sampling time points, more than one sample was taken from some individual patients. Considering that most patients with more than one sample had a different state of disease activity at the different sampling time points, each sample was analyzed as an individual data point.

Further groups were patients with primary food allergy (PFA, *n* = 11), secondary pollen-associated food allergy (SFA, *n* = 25) and *Clostridioides difficile* infection (CDI, *n* = 9). The control group (CON, *n* = 78) consisted of healthy controls (HC, *n* = 37), disease controls without any form of inflammatory gastrointestinal disease (DC, *n* = 13) and patients with irritable bowel syndrome (IBS, *n* = 28). For detailed information on the study groups, see Table 1, Table 2 and the method section.

### 3.2. fECP and fCal Concentration in Controls and Patient Groups

fECP was detectable in 87% (68/78) of the control group samples (detection limit 20 µg/kg). The median fECP level was 124 µg/kg. fECP levels in HC, DC and patients with IBS were not statistically different (median 103, 122 and 147 µg/kg, respectively; Table 3 and Figure S1). Compared to controls, fECP levels were significantly elevated in patients with PFA (median 526 µg/kg), UC (536 µg/kg), CD (502 µg/kg) and CDI (754 µg/kg). fECP was not elevated in patients with SFA (93 µg/kg).

fCal levels in HC, DC and patients with IBS were not statistically different. Although all controls had neither a history of an inflammatory gastrointestinal disease, nor a history of gastrointestinal symptoms, fCal levels between 100 and 250 mg/kg were found in 4.7% (*n* = 3, one HC, one DC, one IBS) and fCal levels above 250 mg/kg were detected in 7.9% (*n* = 5, 3 HC, 2 DC) of controls (Figure S1). fCal was significantly increased in patients with UC, CD and CDI, but not in those with PFA or SFA (Table 3). The highest levels were found in patients with CDI, with a median level of 1305 mg/kg. Four patients with CDI had levels above the detection limit of 16,000 mg/kg.

### 3.3. Association of fECP with Markers of Disease Activity, Disease Phenotype, Medication and Demographics in Patients with IBD 

fECP showed a highly significant correlation with the inflammatory markers fCal and CRP as well as the endoscopic scores in patients with UC (r_s_ = 0.48) and CD (r_s_ = 0.50) (Table 4). However, the correlation of fCal with the endoscopic scores (fCal vs. Mayo, r_s_ = 0.69; fCal vs. SES-CD, r_s_ = 0.64; Table 4) was superior compared to fECP. fECP was only weakly correlated with the clinical score for UC (SCCAI). No significant correlation was found for fECP vs. HBI, the clinical score for CD. A similar picture was observed for the correlations between fCal and clinical scores (Table 4). fECP levels were not associated with clinical factors, such as sex, BMI, IBD type, disease localization, behavior (Montreal classification), perianal disease, presence of upper GI tract involvement, medication with immunomodulators, family history of IBD or smoking status (Table 5). In the univariate analysis age, disease duration and past surgery were negatively associated with fECP, whereas steroid and anti-TNF treatment was positively associated with fECP. In the multivariate analysis, including fCal as a marker of disease activity, only age and fCal remained significantly associated with fECP levels (Table 5). It has to be noted that fCal in patients with IBD and fECP in controls did not correlate with age.

### 3.4. fECP and fCal as Disease Markers in Different Age Groups

**Figure 1:**
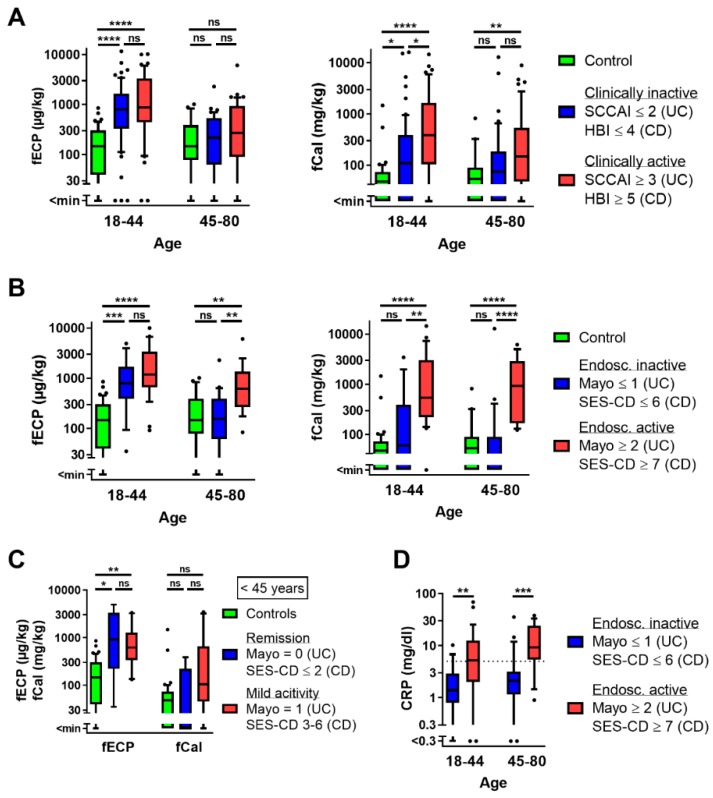
Age dependency of fECP and fCal. (**A**) fECP and fCal in controls (<45 years, *n* = 52; ≥45 years, *n* = 26) and clinically inactive (<45 years, *n* = 60; ≥45 years, *n* = 42) and active (<45 years, *n* = 44; ≥45 years, *n* = 47) patients with IBD according to the clinical scores (SCCAI for UC, HBI for CD). (**B**) fECP and fCal in controls (<45 years, *n* = 52; ≥45 years, *n* = 26) and endoscopically inactive (<45 years, *n* = 15; ≥45 years, *n* = 22) and active (<45 years, *n* = 29; ≥45 years, *n* = 17) patients with IBD according to the endoscopic scores (Mayo for UC, SES-CD for CD). (**C**) fECP and fCal in controls (*n* = 52), patients with IBD in complete endoscopic remission (*n* = 5) and patients with IBD with mild endoscopic activity (*n* = 10) below the age of 45 years. (**D**) Serum CRP levels in endoscopically inactive (<45 years, *n* = 15; ≥45 years, *n* = 22) and active (<45 years, *n* = 28; ≥45 years, *n* = 17) patients with IBD. Kruskal–Wallis test followed by Dunn’s post-hoc comparisons (three groups) or Mann–Whitney U test (two groups); *, *p* < 0.05; **, *p* < 0.01; ***, *p* < 0.001; ****, *p* < 0.0001.

Because fECP is inversely correlated with age in patients with UC and CD, we next analyzed fECP and fCal in controls and patients with IBD in different age groups (18–44 years and 45–80 years). Below the age of 45 years, fECP was elevated in clinically and endoscopically inactive as well as in clinically and endoscopically active patients with IBD compared to controls. There were no significant differences between clinically or endoscopically inactive vs. active patients. In older patients with IBD (45–80 years), fECP was only significantly increased in endoscopically active patients (Figure 1A,B). In contrast, the pattern of fCal levels was very similar between younger and older patients. fCal was significantly elevated in clinically and endoscopically active patients compared to controls. In clinically and endoscopically inactive patients, only minor differences were found compared to controls without IBD (Figure 1A,B). 

Endoscopically inactive IBD patients in Figure 1B were defined as patients in the status of endoscopic remission (Mayo = 0, SES-CD ≤ 2) or with low endoscopic activity (Mayo = 1, SES-CD = 3–6). Figure 1C shows that fECP levels in young patients with IBD were equally increased in patients with endoscopic remission and low activity, while there was only a non-significant tendency for fCal elevation in patients with low endoscopic activity. 

Serum CRP levels were elevated above the upper limit of normal (ULN) (5 mg/dL) in 4/37 (10.8%) of endoscopically inactive patients and in 27/46 (60.9%) of patients with endoscopic activity. As for fCal, the pattern of serum CRP levels was similar in younger and older patients (Figure 1D). 

The same analyses as shown in Figure 1 for all patients with IBD were performed for patients with CD and UC separately, and results are shown in Figures S2 and S3. The overall results show no major differences between patients with CD and UC. 

ROC analyses and sensitivity/specificity calculations show that both, fECP and fCal, can differentiate between endoscopically active patients with IBD and controls as well as between endoscopically active and inactive patients with IBD (Figure S4 and Table S2). However, the overall performance of fCal in these comparisons is better than for fECP. The area under the curve (AUC) in the ROC analyses and sensitivity/specificity/accuracy (cut-off) for the differentiation between endoscopically active and inactive patients with IBD was 0.88 and 98%/76%/87% (118 mg/kg) for fCal and 0.77 and 80%/65%/73% (451 µg/kg) for fECP, respectively. 

Both fCal and fECP had a low accuracy differentiating between clinically active and inactive patients with IBD. In contrast to fCal, fECP could differentiate between controls and patients with IBD <45 years regardless of clinical or endoscopic activity with accuracies above 80% and AUC levels above 0.8 (Figure S4 and Table S2). The best cut-off levels were between 309 and 373 µg/kg (Supplementary Table S2). Subgroup analysis did not show major differences between patients with UC and CD in the ROC analyses and sensitivity/specificity calculations (Figure S4).

These results suggest that fCal is the superior biomarker for inflammatory activity in patients with IBD, whereas fECP is a good diagnostic marker for young patients with IBD independent of clinical and inflammatory activity. 

### 3.5. fECP Is a Prognostic Biomarker in Young Patients with IBD

Next, we tested whether fECP could predict disease progression. To measure disease progression, we analyzed the time to first treatment modification (start or change of immunosuppressants or biologics) or surgery. Clinical follow-up data was available for 202/212 visits (95.3%) of patients with IBD. The median (IQR) follow-up time was 48 (38–54) months. The total event rate was 94/202 (46.5%). The first event was treatment modification in 77/202 (38.1%) or surgery in 17/202 (8.4%).

Furthermore, in patients with a baseline fCal of <250 mg/kg, the time to the first fCal measurement ≥250 mg/kg was documented. fCal measurements were performed as clinically required. fCal follow-up was available for 119/130 visits (91.5%). The median (IQR) frequency of fCal analyses in this patient group was 1.9/year (1.4–3.1). The total event rate (fCal measurement ≥250 mg/kg) was 57/119 (47.9%).

**Figure 2:**
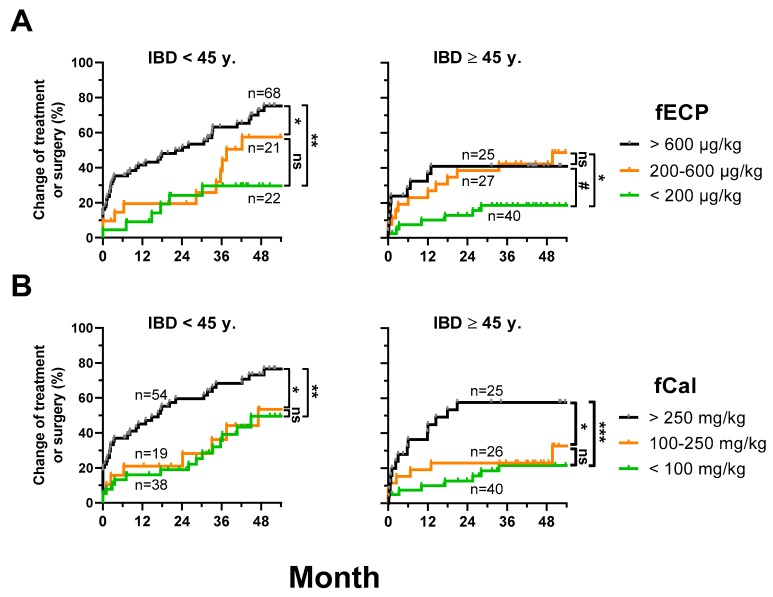
fECP as a predictive marker in IBD. Inverse Kaplan–Meier curves for time to treatment modification (only immunosuppressants and biologics) or surgery during follow up depending on (**A**) different levels of fECP and (**B**) different levels of fCal at baseline. *p*-values were calculated with log-rank (Mantel–Cox) test. ns (non-significant), *p* > 0.07; #, *p* ≤ 0.07; *, *p* < 0.05; **, *p* < 0.01; ***, *p* < 0.001.

Disease progression was significantly different depending on the baseline fECP levels. In patients <45 years, the event rates (treatment modification or surgery) were significantly higher with a baseline fECP ≥600 µg/kg than in patients with a baseline fECP <200 µg/kg or 200–600 µg/kg. In patients ≥45 years, differences in the event rates were less pronounced. Patients with a baseline fECP <200 µg/kg had the lowest event rates, whereas the groups 200–600 µg/kg and ≥ 600 µg/kg had slightly higher rates (Figure 2A). 

Baseline fCal was also an indicator of disease progression. Low event rates were found in patients with a baseline fCal <100 mg/kg and 100–250 mg/kg, while there was no significant difference between these groups. Much higher event rates were documented in patients with a baseline fCal ≥250 mg/kg. There were no obvious differences in patients <45 years and ≥45 years (Figure 2B).

**Figure 3:**
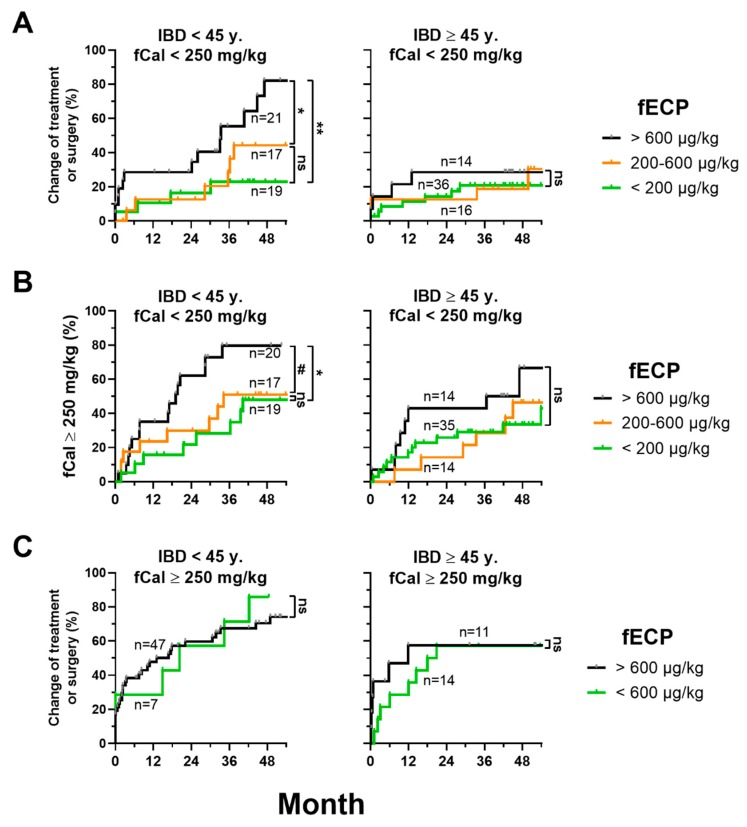
fECP is a predictive marker in young patients with IBD and with low inflammatory activity. (**A**) Inverse Kaplan–Meier curves as in Figure 2 including only patients with fCal levels <250 mg/kg at baseline. (**B**) Inverse Kaplan–Meier for the time to the first fCal measurement ≥250 mg/kg during follow-up including the same patients as in (A) depending on different levels of fECP at baseline. (**C**) Inverse Kaplan–Meier for treatment modification or surgery depending on different fECP levels at baseline including only patients with fCal levels ≥250 mg/kg at baseline. *p*-values were calculated with log-rank (Mantel–Cox) test. ns (non-significant), *p* > 0.07; #, *p* ≤ 0.07; *, *p* < 0.05; **, *p* < 0.01; ***, *p* < 0.001.

Since the difference between normal fCal (<100 mg/kg) and borderline elevated fCal (100–250 mg/kg) was not indicative for disease progression, we analyzed whether fECP levels could be of prognostic value in this group of patients. We found that in patients <45 years and baseline fCal <250 mg/kg, fECP could indeed predict disease progression. In this subgroup, patients with a baseline fECP of ≥600 µg/kg had significantly higher event rates (time to treatment modification/surgery as well as time to first fCal measurement >250 mg/kg) than patients with a baseline fECP <200 µg/kg or 200–600 µg/kg. In contrast, there was no prognostic value of fECP in older patients ≥45 years (Figure 3A,B). Disease progression in patients with a baseline >250 mg/kg was independent of baseline fECP (Figure 3C).

## 4. Discussion 

In the present study, the diagnostic value of fECP in patients with IBD was analyzed and compared to fCal. We found that fECP correlates with endoscopic activity and hence could serve as a biomarker in IBD. In contrast to fCal, fECP was elevated in the majority of patients with IBD below the age of 45 years regardless of clinical and endoscopic activity and could therefore serve as a diagnostic marker in young patients with IBD in remission. Furthermore, we found that fECP is a prognostic marker in young patients with IBD, predicting an inflammatory relapse during follow-up and the need for treatment modification or surgery. 

The fact that fecal eosinophil granular proteins are elevated in patients with IBD has been shown by several recent studies [35,36,37,38,39,40,41]. However, these studies were performed in smaller cohorts than in our study. Some of the studies reported a correlation of fEPX or fECP with clinical activity in patients with UC [36,37,41] and CD [35,37], whereas one study did not find such associations [38]. fEPX or fECP levels correlated with the levels of fecal neutrophil-derived proteins fCal [40], myeloperoxidase [40] and lactoferrin [37].

We found a moderate correlation of fECP with clinical activity in patients with UC, but no correlation with clinical activity in CD. However, this pattern was not unique to fECP considering that a similar picture was found for fCal and serum CRP. The fact that inflammatory biomarkers poorly correlate with clinical activity scores, particularly in patients with CD, has also been reported by other studies [6,43]. The most likely causes of this effect are symptoms related to non-inflammatory structural changes of the intestine such as scarring, strictures, adhesions in later stages of the disease or presence of bile acid stricture in post-surgical patients.

One study reported a correlation of fEPX and fECP levels with histological scores in patients with UC [41]. However, to the best of our knowledge, a correlation of endoscopic IBD scores with fecal eosinophil-derived biomarkers has not yet been performed. We found a highly significant correlation of fECP levels with the endoscopic Mayo Score for UC and the SES-CD for CD. Nevertheless, the discriminant accuracy to differentiate between endoscopically active and inactive patients was lower for fECP than for fCal. A combination of fECP and fCal did not increase diagnostic accuracy either.

The lower sensitivity and specificity of fECP in discriminating endoscopically active and inactive patients with IBD was partially related to the fact that in young patients below the age of 45 years, fECP was also elevated during endoscopic remission or low endoscopic activity, which was not the case for fCal. Consequently, fECP could discriminate between controls and young clinically and endoscopically inactive patients with IBD with an accuracy above 80%. Therefore, fECP might be a good screening biomarker in young adult patients, particularly in those with intermittent symptoms. A recent study suggested that colonic eosinophilia is an early histological sign for IBD in children. Patients with IBD who had an initial inconclusive endoscopy but a later confirmatory diagnosis of IBD had about three times more colonic eosinophils than patients with a diagnosis of functional abdominal disorders [44].

The reasons why eosinophils seem to be more active in younger patients and also during remission are not known and, as yet, have been largely uninvestigated. It has been reported that colonic eosinophilia is more prevalent in younger than in older children diagnosed with IBD or other colonic diseases [45,46]. Furthermore, tissue eosinophilia seems to be a dominant feature of subclinical histological inflammation for patients with IBD in remission [47], which might be more prevalent in young patients with IBD.

However, like fCal, it must be considered that fECP is not specific for IBD, since increased levels are also found in patients with food allergy [38,48,49,50,51] or infectious disease [52], which was confirmed in our study. Increased levels of fECP are also present in patients with collagenous colitis [32] and eosinophilic enterocolitis [28]. Unlike in patients with a primary food allergy, we did not detect elevated fECP levels in patients with pollen-associated food allergy. These patients suffer from adverse reactions to pollen cross-reactive food allergens that are often unstable towards gastric acid and digestive enzymes. Consequently, allergic symptoms in these patients occurred mostly in the upper part of the GI system (oral allergy syndrome or eosinophilic esophagitis) or at the systemic level (skin or respiratory symptoms) [53]. 

Estimating disease prognosis in IBD is a major challenge for clinicians. An aggressive early treatment might improve the long-term prognosis of some patients. However, applying a “top–down” treatment to all patients with IBD may lead to the overtreatment of many patients with a mild disease course. Another challenge is the timing of therapy withdrawal during remission, and most guidelines do not give specific recommendations. Some clinical features indicate a more complicated disease course in patients with CD or UC, and these features include young age, extensive bowel involvement, presence of perianal disease at diagnosis, initial treatment with systemic steroids, and co-occurrence of primary sclerosing cholangitis as well as some biomarkers, such as the presence of anti-microbial antibodies ASCA [25,54]. Elevated fCal levels and persistent histological inflammation during remission have been shown to indicate increased risk for disease relapse [14,15,16,17,18,19,47,55,56]. Noteworthy, tissue eosinophilia is among the major histological features that are associated with high risk for recurrence [47]. Functional studies with isolated eosinophils showed that colon-derived eosinophils are activated during the remission phase of UC [57].

We analyzed the time to change or start of treatment with immunosuppressants or biologics or requirement of surgery as surrogate markers for disease progression. As expected, elevated fCal levels at baseline were associated with increased event rates. Interestingly, no differences were found in the groups with fCal levels <100 mg/kg and 100–250 mg/kg. 

In several recent studies, it has been shown that fCal levels measured in patients with IBD during remission can predict disease relapse during follow-up [14,15,16,17,18,19]. The best cut-off values varied in the different studies between 50 and 300 mg/kg, most likely because different assays were used, and slightly different patient cohorts were studied. The major difference in our analysis compared to the mentioned studies was that we did not focus on clinical relapse, but disease progression defined as above.

Higher fECP levels were also associated with increased rates of treatment change or surgery. Most importantly, in patients <45 years with low inflammation as defined by fCal levels <250 mg/kg, fECP could define patients as low or high risk for requiring treatment modification, surgery or inflammatory relapse, indicated by fCal levels >250 mg/kg during follow-up. Here, fECP had a clear additional value to fCal, since, in this subgroup, fCal (<100 mg/kg vs. 100–250 mg/kg) was not sufficient for further stratification.

A limitation to this study was the retrospective design and that clinical scores and steroid treatment were not considered for the follow-up analysis, because of potential incomplete documentation. Short-term steroid therapy was not always documented in the patient’s reports and sometimes initiated by the patient’s General Practitioner.

In summary, we found that analyzing fecal fECP is of additional value for monitoring young adult patients with IBD. Most importantly, it could be used as a diagnostic tool and as a biomarker indicating disease progression or relapse probability. Further prospective studies analyzing fecal eosinophil proteins as biomarkers in IBD also involving children and adolescents would be of interest. One important question could be whether fECP could predict the risk of disease relapse after treatment withdrawal or surgery. If this was the case, fECP could stratify patients for the requirement of continuing remission-maintaining or starting prophylactic treatment. 

## 5. Conclusions

fECP is significantly correlated with fCal, and both markers correlate with endoscopic activity and therefore serve as good biomarkers in IBD.

Both fECP and fCal correlate poorly with clinical activity scores.

Irrespective of clinical and endoscopic activity, fECP was elevated in patients <45 years with IBD. Therefore, it can serve as a diagnostic marker during clinical remission and differentiate young patients with IBD from patients with IBS.

In patients with IBD <45 years with low inflammatory activity, elevated fECP indicated the need for treatment modification or surgery during follow up and can therefore serve as a prognostic marker in young patients with IBD.

## Figures and Tables

**Table 1:** Study participants.

	*n*	Age Mean (±SD)	Female Sex *n* (%)	BMI (kg/m^2^) Mean (±SD)
Controls (HC + DC + IBS)	78	38 ± 15	45 (58)	24 ± 5
Healthy control (HC)	37	33 ± 13	21 (57)	22 ± 2
Disease control (DC) ^1^	13	57 ± 13	5 (38)	24 ± 4
Irritable bowel syndrome (IBS)	28	37 ± 13	19 (68)	25 ± 6
Secondary food allergy (SFA) ^2^	25	41 ± 14	17 (68)	25 ± 4
Primary food allergy (PFA)	11	41 ± 15	7 (64)	25 ± 6
Ulcerative colitis (UC)	53	43 ± 13	25 (47)	27 ± 5
Crohn’s disease (CD)	97	42 ± 14	51 (53)	25 ± 6
*Clostridioides difficile* infection (CDI)	9	70 ± 12	5 (56)	29 ± 5

**^1^** Main diagnoses: liver disease (7), diabetes (1), thyroid disease (1), gastritis (2), cured gastrointestinal (GI) malignancy (2) and ^2^ pollen-associated food allergy.

**Table 2:** Detailed characteristics of patients with inflammatory bowel disease (IBD).

	Ulcerative Colitis (UC) Patients Visits, Total Samples	*n* (%) 53 69	Crohn’s Disease (CD) Patients Visits, Total Samples	*n* (%) 97 143
Age diagnosis	A1 (<17 years) A2 (17–40 years) A3 (>40 years)	2 (4) 36 (68) 15 (28)	A1 (<17 years) A2 (17–40 years) A3 (>40 years) Not documented	13 (13) 68 (70) 14 (14) 2 (2)
Localization	E1 (proctitis) E2 (left-sided colitis) E3 (pancolitis)	1 (2) 24 (45) 28 (55)	L1 (ileal) L2 (colonic) L3 (ileocolonic) L4* (+ upper GI disease) L4 (isolated upper GI dis.) Not documented	35 (36) 15 (16) 44 (45) 27 (28) 1 (1) 2 (2)
Behavior	P (perianal disease) Previous surgery^1^	9 (17) 1 (2)	B1 (inflammatory) B2 (stricturing) B3 (penetrating) p (perianal disease) Previous surgery^1^	54 (55) 24 (24) 20 (20) 39 (40) 31 (32)
Disease duration years (mean ± SD)	9.7 ± 8.2	53	12.3 ± 10.5	95
Smoking status	Never Former Current	23 (43) 27 (51) 3 (6)	Never Former Current	43 (44) 26 (27) 28 (29)
Family history	IBD negative IBD positive Unknown	39 (74) 11 (21) 3 (6)	IBD negative IBD positive Unknown	75 (77) 19 (20) 3 (3)
Clinical activity Missing: *n* = 19	Inactive (SCCAI ≤ 2) Mild (SCCAI 3–5) Moderate (SCCAI 6–10) Severe (SCCAI ≥ 11)	28 (44) 19 (30) 15 (23) 2 (3)	Inactive (HBI ≤ 4) Mild (HBI 5–7) Moderate (HBI 8–16) Severe (HBI ≥ 17)	74 (57) 22 (17) 31 (24) 2 (2)
Endoscopic activity Not performed: *n* = 129	Inactive (Mayo = 0) Mild (Mayo = 1) Moderate (Mayo = 2) Severe (Mayo = 3)	3 (7) 12 (29) 14 (34) 12 (29)	Inactive (SES-CD 0–2) Mild (SES-CD 3–6) Moderate (SES-CD 7–15) Severe (SES-CD ≥ 16)	14 (33) 8 (19) 14 (33) 6 (14)
Medication Missing: *n* = 0	5-ASA ^2^ Steroids ^3^ Steroids (>10 mg ^4^) Thiopurins Methotrexat Anti-TNF ^5^ Vedolizumab Tacrolimus Antibiotics/Virostatics Probiotics	63 (92) 27 (39) 13 (19) 19 (28) 1 (1) 21 (30) 0 (0) 2 (3) 3 (4) 1 (1)	5-ASA ^2^ Steroids ^3^ Steroids (>10 mg ^4^) Thiopurins Methotrexat Anti-TNF Vedolizumab Tacrolimus Antibiotics/Virostatics Probiotics	40 (28) 32 (22) 11 (8) 34 (24) 1 (1) 56 (39) 1 (1) 1 (1) 7 (5) 0 (0)
CRP (mg/dL) Median (IQR) ≥5 mg/dL	2.1 (0.9–6.9)	68 25 (37)	2.3 (0.7–5.9)	140 39 (28)

* patients with L1/ L2 or L3, combined with L4 (upper GI disease), ^1^ IBD-related surgery without perianal surgery, ^2^ including topical 5-aminosalicylic acid (ASA), ^3^ budesonide and topical steroids, ^4^ prednisolon/prednison equivalent, and ^5^ tumor necrosis factor (TNF). CRP: C-reactive protein; HBI: Harvey-Bradshaw Index; SCCAI: Simple Clinical Colitis Activity Index; SES-CD: Simple Endoscopic Score for CD.

**Table 3:** Fecal eosinophil cationic protein (ECP) (µg/kg) and calprotectin (mg/kg) in different patient groups.

Group	*n*	fECP (µg/kg) Median (IQR)	*p* ^1^	fCal (mg/kg) Median (IQR)	*p* ^1^
**CON**	78	**124** (42–302)		**52** (<39–75)	
**HC**	37	**103** (38–288)		**51** (<39–75)	
**DC**	13	**122** (64–648)	0.40	**53** (<39–103)	0.99
**IBS**	28	**147** (<20–266)	0.89	**52** (<39–68)	0.37
**SFA**	25	**93** (<20–1029)	0.71	**<39** (<39–88)	0.72
**PFA**	11	**526** (107–1085)	**0.0064**	**63** (<39–162)	0.71
**UC**	69	**536** (148–1230)	**<0.0001**	**186** (43–1235)	**<0.0001**
**CD**	143	**502** (130–1145)	**<0.0001**	**135** (<39–384)	**<0.0001**
**CDI**	9	**754** (246– >12,000)	**0.0052**	**1305** (536– >16,000)	**<0.0001**

CON, controls (HC + DC + IBS); HC, healthy controls; DC, disease controls; IBS, irritable bowel syndrome; SFA, secondary (pollen-associated) food allergy; PFA, primary food allergy; UC, ulcerative colitis; CD Crohn’s disease; CDI, Clostridioides difficile infection. ^1^ Mann–Whitney U test; DC/IBS vs. HC; PFA/UC/CD/CDI vs. CON. Bold p: highly significant.

**Table 4:** Correlations of fECP with different disease activity markers.

Comparison/Spearman Corr.	Ulcerative Colitis			Crohn’s Disease		
	r_s_	*p*	n	r_s_	*p*	n
fECP vs. Mayo/SES-CD	0.48	0.001	41	0.50	0.001	42
fECP vs. fCal	0.66	<0.001	69	0.46	<0.001	143
fECP vs. CRP	0.31	0.010	68	0.36	<0.001	140
fECP vs. SCCAI/HBI	0.32	0.011	64	0.01	0.88	129
fCal vs. Mayo/SES-CD	0.69	<0.001	41	0.64	<0.001	42
fCal vs. fECP	0.66	<0.001	69	0.46	<0.001	143
fCal vs. CRP	0.54	<0.001	68	0.50	<0.001	140
fCal vs. SCCAI/HBI	0.40	0.001	64	0.14	0.11	129

r_s_ = < 0.3; r_s_ = 0.30–0.44; r_s_ = 0.45–0.59; r_s_ ≥ 0.60.

**Table 5:** Univariate and multivariate analyses of factors with potential influence on fECP.

	Univariate Analysis ^1^	Multivariate Analysis ^2^
fCal	**<0.0001 (+)**	**<0.0001**
Age	**<0.0001 (−)**	**<0.0001**
Sex	0.42	-
BMI	0.58	-
Disease (CD vs. UC)	0.72	-
Disease duration	**<0.0001 (−)**	0.57
Localization UC ^3^	0.74	-
Localization CD ^3^	0.29	-
Behavior CD ^3^	0.39	-
Upper GI disease CD	0.11	-
Perianal disease	0.079	-
Surgery	**0.015 (−)**	0.82
Steroids	**0.009 (+)**	0.62
Immunomodulators	0.16	-
Anti-TNF	**0.016 (+)**	0.67
Family history	0.58	-
Smoking status	0.19	-

^1^ Spearman correlation, Mann–Whitney U or Kruskal–Wallis test. ^2^ Linear logistic regression using Log10(fECP) as the dependent variable and Log10(fCal), age, disease duration, surgery, steroids and anti-TNF as the independent variables. ^3^ Montreal classification. (+) Positive association; (−) Negative association. Bold: highly significant association.

## Supplementary Materials

The supplementary materials are available online at https://www.mdpi.com/2077-0383/8/12/2025/s1.
