# Supplementary material for: Fecal Eosinophil Cationic Protein Is a Diagnostic and Predictive Biomarker in Young Adults with Inflammatory Bowel Disease
Source: J Clin Med. 2019 Nov 20;8(12):2025. doi: 10.3390/jcm8122025 (PMC6947361; doi:10.3390/jcm8122025)
Supplement: Supplementary file 1 [file jcm-08-02025-s001.pdf]

Supplementary data

Suppl. Figure 1

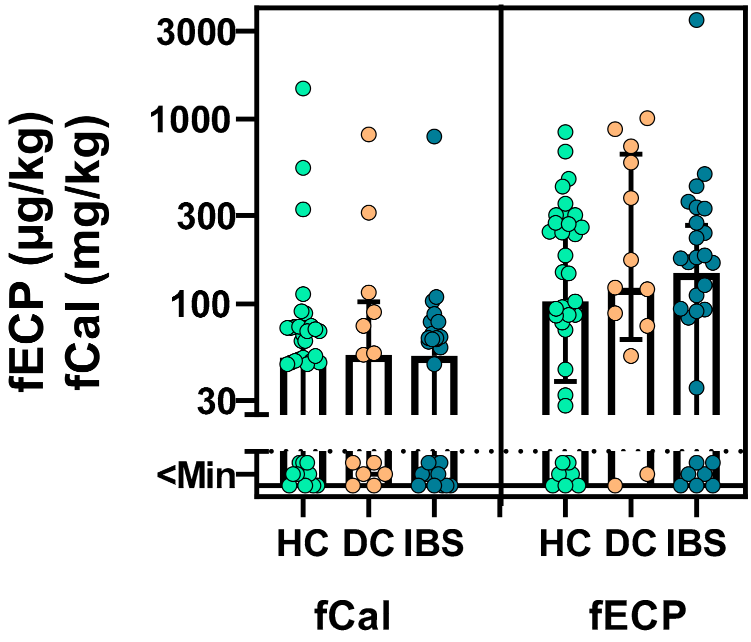

Suppl. Figure 1. fECP and fCal are similarly distributed in the different control groups (HC, healthy controls; DC, disease control; IBS, irritable bowel syndrome). Shown are single data points (dots), median (bars) and IQR (error bars).
Because of the similar data distribution in the three groups, pooled data were used as s single control group throughout the study.

Suppl. Figure 2

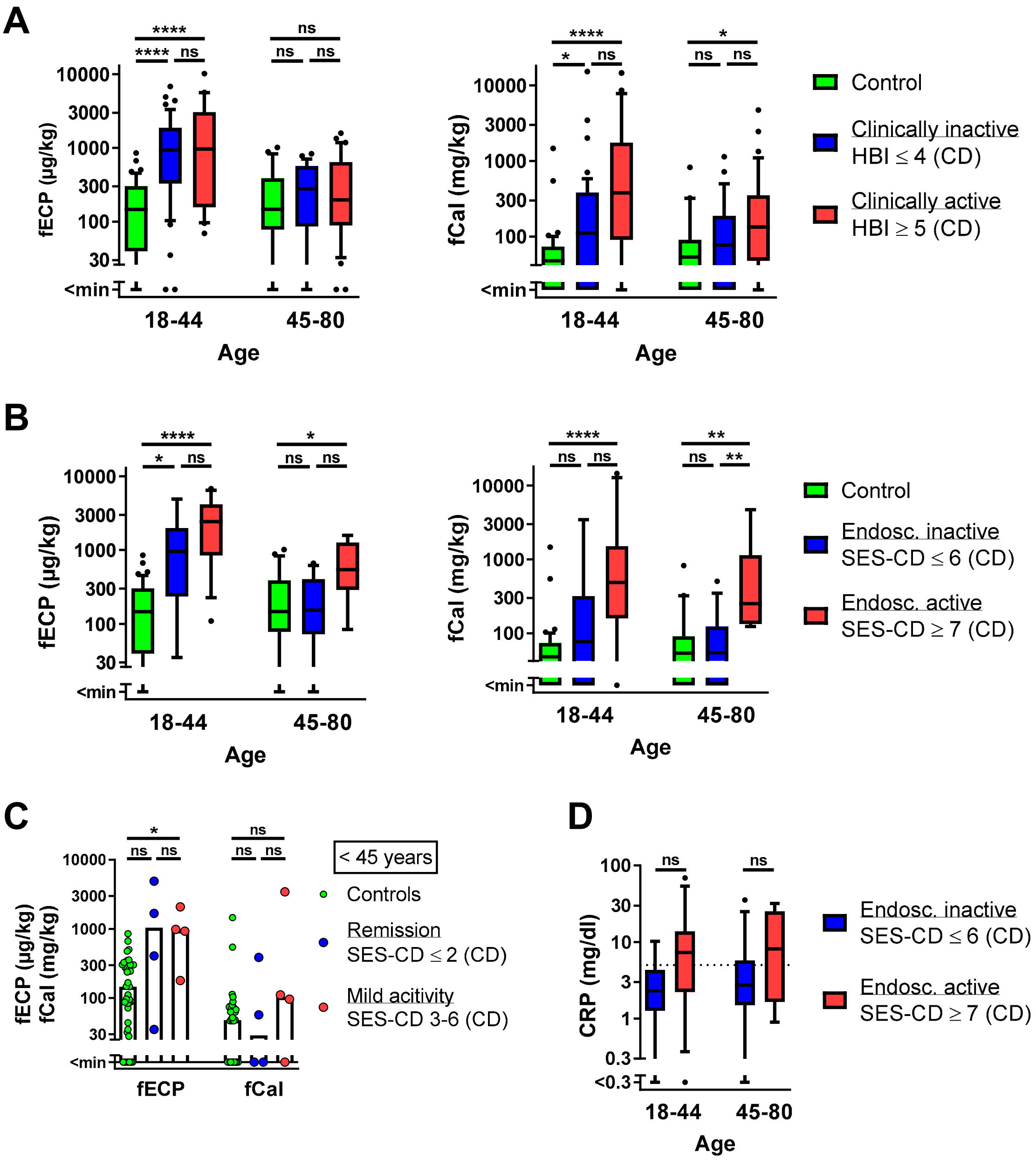

Suppl. Figure 2. Age dependency of fECP and fCal in CD patients. (A) fECP and fCal in controls (<45 y., n = 52; ≥45 y., n = 26) and clinically inactive (<45 y., n = 45; ≥45 y., n = 29) and active (<45 y., n = 23; ≥45 y., n = 43) CD patients according to the clinical score HBI. **(B)** fECP and fCal in controls (<45 y., n = 52; ≥45 y., n = 26) and endoscopically inactive (<45 y., n = 8; ≥45 y., n = 14) and active (<45 y., n = 12; ≥45 y., n = 8) CD patients according to the endoscopic score SES-CD. **(C)** fECP and fCal in controls (n = 52), CD patients in complete endoscopic remission (n = 4) and CD patients with mild endoscopic activity (n = 4) below the age of 45 years (bars represent median). (D) Serum CRP levels in endoscopically inactive (<45 y., n = 8; ≥45 y., n = 14) and active (<45 y., n = 12; ≥45 y., n = 8) CD patients. Kruskal-Wallis test followed by Dunn's post-hoc comparisons (3 groups) or Mann-Whitney-U test (2 groups); *, p <0.05; **, p <0.01; ***, p <0.001; ****, p <0.0001.

Suppl. Figure 3

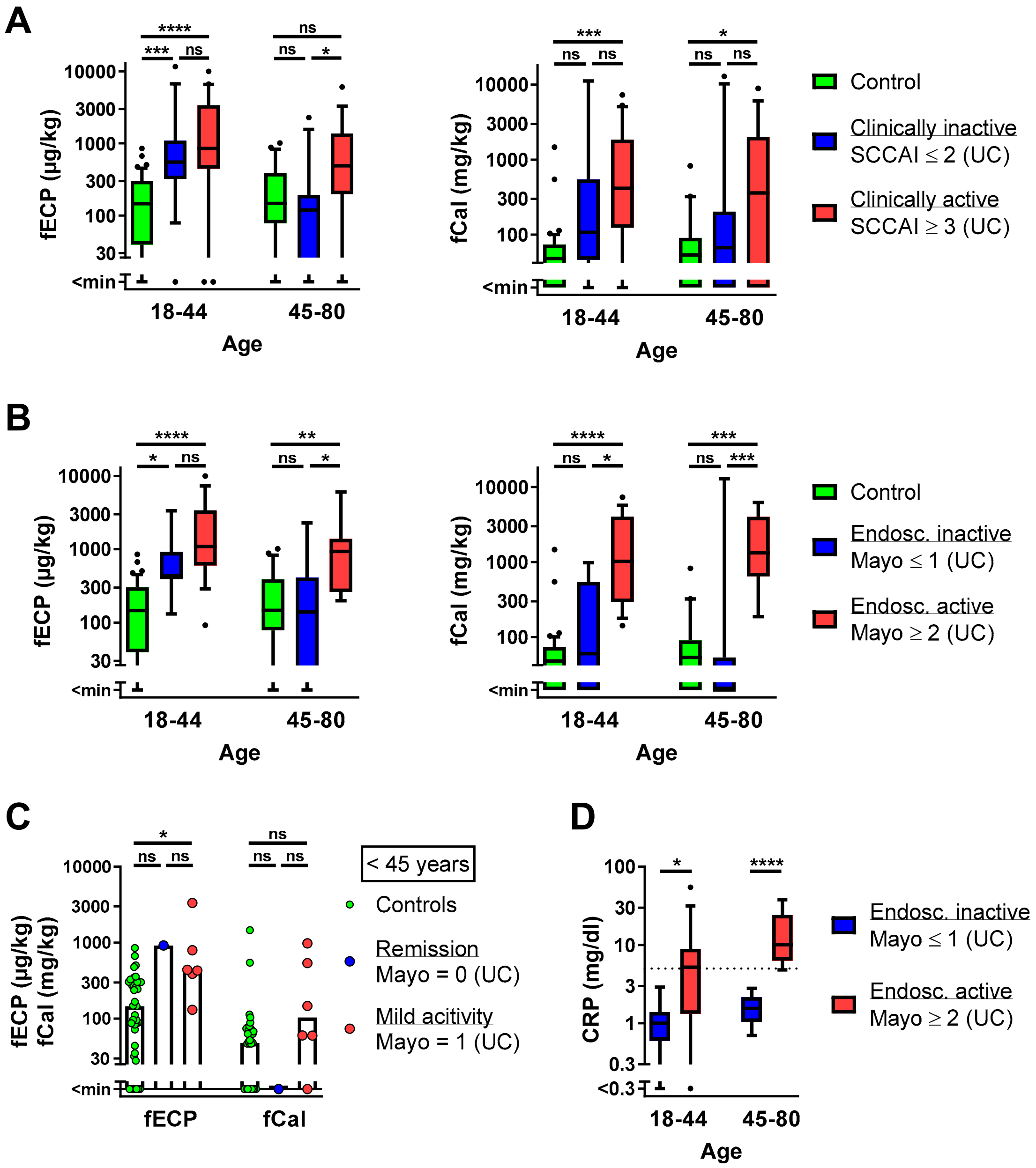

Suppl. Figure 3. Age dependency of fECP and fCal in UC patients. (A) fECP and fCal in controls (<45 y., n = 52; ≥45 y., n = 26) and clinically inactive (<45 y., n = 15; ≥45 y., n = 13) and active (<45 y., n = 21; ≥45 y., n = 15) UC patients according to the clinical score SCCAI. **(B)** fECP and fCal in controls (<45 y., n = 52; ≥45 y., n = 26) and endoscopically inactive (<45 y., n = 7; ≥45 y., n = 8) and active (<45 y., n = 17; ≥45 y., n = 9) UC patients according to the endoscopic Mayo score. **(C)** fECP and fCal in controls (n = 52), UC patients in complete endoscopic remission (n = 1) and CD patients with mild endoscopic activity (n = 6) below the age of 45 years (bars represent median). (D) Serum CRP levels in endoscopically inactive (<45 y., n = 7; ≥45 y., n = 8) and active (<45 y., n = 16; ≥45 y., n = 9) UC patients. Kruskal-Wallis test followed by Dunn's post-hoc comparisons (3 groups) or Mann-Whitney-U test (2 groups); *, p <0.05; **, p <0.01; ***, p <0.001; ****, p <0.0001.

Suppl. Figure 4

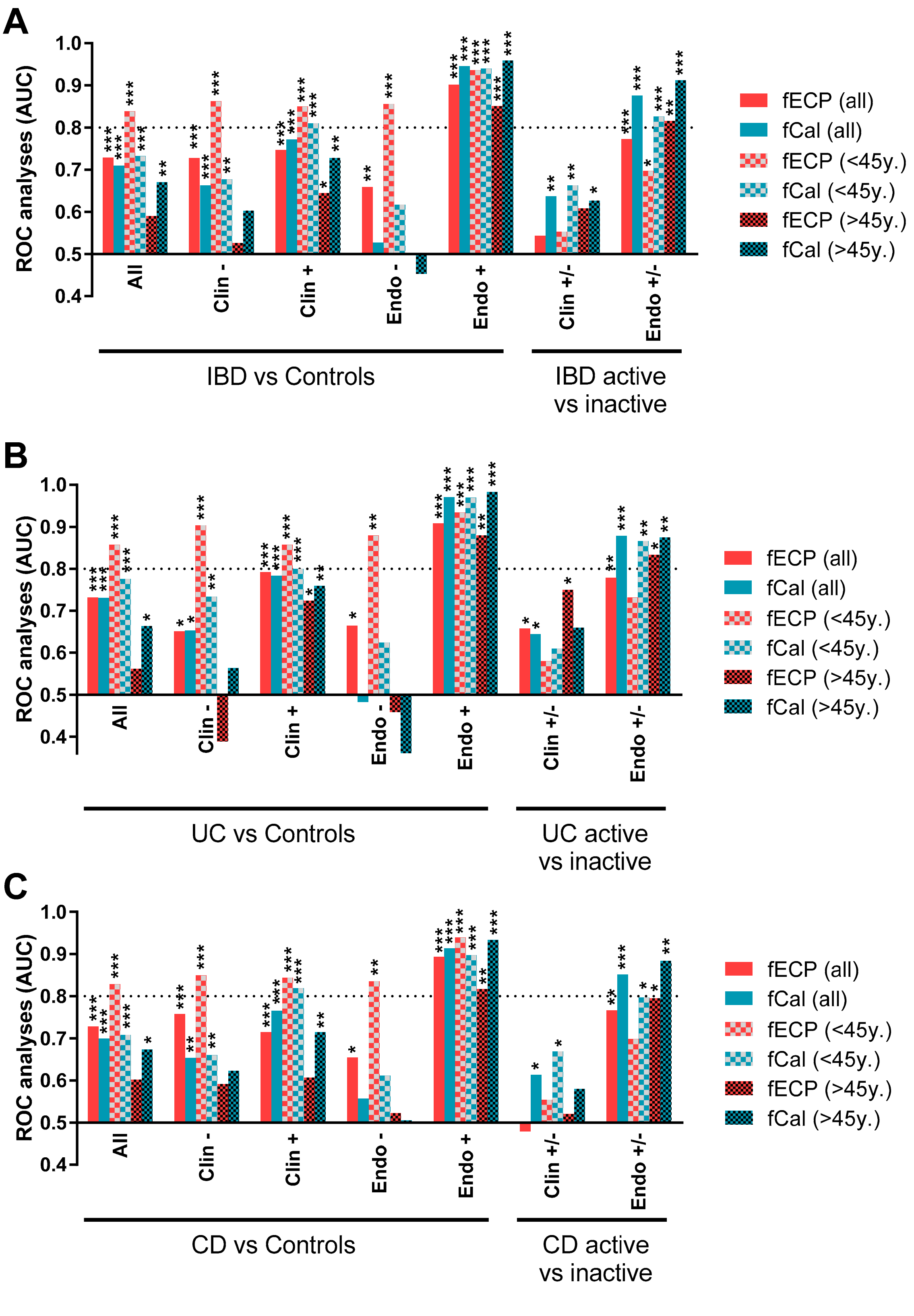

Suppl. Figure 4. ROC analyses. Graphs show the area under the curve (AUC) values. Statistical significances are depicted by asterix above the bars. *, p <0.05; **, p <0.01; ***, p <0.001. Definitions: Clin-, clinically inactive (SCCAI ≤ 2 / HBI ≤ 4); Clin+ (SCCAI ≥3 / HBI ≥5); Endo- (Mayo ≤1 / SES-CD ≤6); Endo+ (Mayo ≥2 / SES-CD ≥7); 45y; age 45 years. **(A)** All IBD patients (vs. controls). **(B)** Only ulcerative colitis (vs. controls). **(C)** Only Crohn's disease (vs. controls).

Suppl. Table 1. Best cut-off values and related sensitivity, specificity and accuracy for fECP and fCal

			Best cut-off (highest accuracy)		Sensitivity/specificity (accuracy[1])	
		n	**fECP** (µg/kg)	**fCal** (mg/kg)	**fECP** (%)	**fCal** (%)
IBD vs. control	**All**	212/78	477	118	52/90 (71)	57/92 (75)
	**All <45y**	115/52	373	90	**72/90 (81)**	66/89 (77)
	**All ≥45y**	97/26	247	118	50/73 (61)	54/89 (71)
	**Clinically inactive**	102/78	309	94	62/80 (71)	51/87 (69)
	**Clinically inactive <45y**	59/52	309	93	**81/83 (82)**	58/89 (73)
	**Clinically inactive ≥45y**	43/26	200	118	51/65 (58)	40/89 (64)
	**Clinically active**	91/78	375	119	60/86 (73)	**70/92 (81)**
	**Clinically active <45y**	44/52	373	128	**80/90 (85)**	**75/94 (85)**
	**Clinically active ≥45y**	47/26	247	98	55/73 (64)	70/85 (77)
	**Endoscopically inactive**	37/78	358	94	49/85 (67)	32/87 (60)
	**Endoscopically inactive <45y**	15/52	373	93	**80/90 (85)**	47/89 (68)
	**Endoscopically inactive ≥45y**	22/26	128	98	59/50 (55)	23/85 (54)
	**Endoscopically active**	46/78	448	120	**80/89 (84)**	**98/92 (95)**
	**Endoscopically active <45y**	29/52	410	123	**90/90 (90)**	**97/94 (95)**
	**Endoscopically active ≥45y**	17/26	247	120	**88/73 (81)**	**100/89 (94)**
IBD subgroups	**Clinically active/inactive**	91/102	592	122	52/61 (56)	70/56 (63)
	**Clinically active/inactive <45y**	44/59	2124	143	39/80 (59)	75/56 (66)
	**Clinically active/inactive ≥45y**	47/43	834	122	28/93 (63)	66/63 (64)
	**Endosc. active/inactive**	46/37	451	118	80/65 (73)	**98/76 (87)**
	**Endosc. active/inactive <45y**	29/15	451	122	90/47 (68)	**97/67 (82)**
	**Endosc. active/inactive ≥45y**	17/22	183	114	94/64 (79)	**100/82 (91)**

[1] corrected for different group sizes. Accuracy >80 % in bold.
